# Dysphagia in the intensive care unit: epidemiology, mechanisms, and clinical management

**DOI:** 10.1186/s13054-019-2400-2

**Published:** 2019-03-28

**Authors:** Patrick Zuercher, Céline S. Moret, Rainer Dziewas, Joerg C. Schefold

**Affiliations:** 1 Department of Intensive Care Medicine, Inselspital, Bern University Hospital, University of Bern, 3010 Bern, CH Switzerland; 2 Department of Neurology, University Hospital Münster, Münster, Germany

**Keywords:** Deglutition disorder, ICU-acquired swallowing dysfunction, ICU-acquired weakness, Critical illness, Sepsis

## Abstract

Dysphagia may present in all critically ill patients and large-scale clinical data show that e.g. post-extubation dysphagia (PED) is commonly observed in intensive care unit (ICU) patients. Recent data demonstrate that dysphagia is mostly persisting and that its presence is independently associated with adverse patient-centered clinical outcomes. Although several risk factors possibly contributing to dysphagia development were proposed, the underlying exact mechanisms in ICU patients remain incompletely understood and no current consensus exists on how to best approach ICU patients at risk.

From a clinical perspective, dysphagia is well-known to be associated with an increased risk of aspiration and aspiration-induced pneumonia, delayed resumption of oral intake/malnutrition, decreased quality of life, prolonged ICU and hospital length of stay, and increased morbidity and mortality. Moreover, the economic burden on public health care systems is high.

In light of high mortality rates associated with the presence of dysphagia and the observation that dysphagia is not systematically screened for on most ICUs, this review describes epidemiology, terminology, and potential mechanisms of dysphagia on the ICU. Furthermore, the impact of dysphagia on affected individuals, health care systems, and society is discussed in addition to current and future potential therapeutic approaches.

## Background

Dysphagia including post-extubation dysphagia (PED) is a concern in hospitalized patients on intensive care units (ICUs). Earlier studies, which were mostly limited by study design, patient selection, and/or limited patient numbers [1–6], reported conflicting and inconsistent results regarding the incidence of post-extubation dysphagia. In fact, incidence rates ranged from 3 to 62% [7]. Following systematic screening post-extubation, we recently published the largest prospective observational study on PED and observed that the PED incidence in unselected emergency ICU admission was 18.3% [8]. Further, PED persisted until ICU discharge in > 80% of cases and > 60% of patients with impaired deglutition on ICU remained dysphagic at hospital discharge [8]. Importantly, the presence of PED had an impact on morbidity and mortality, with an excess 90-day all-cause mortality rate of 9.2% [8].

In general medical populations, the overall burden of dysphagia on public health care system is considered high. Dysphagia-associated complications include increased risk for aspiration, aspiration-induced pneumonia [6, 9–26], delayed resumption of oral intake/malnutrition [3, 10–13, 27, 28], decreased quality of life [21, 27], prolonged ICU and/or hospital length of stay [3, 8, 11, 14, 29], and increased morbidity and mortality [3, 6, 8, 9, 13, 27, 30–33].

Considering that post-extubation dysphagia is not routinely screened for in most ICUs [34], maybe due to limited awareness, PED appears a rather poorly recognized health care problem. Years following the latest systematic reviews on incidence and mechanisms of swallowing disorders in critically ill ICU patients [4, 7, 35], we embarked to update respective available data in the context of dysphagia epidemiology, potential mechanisms leading to dysphagia, screening approaches, and current and future treatment modalities.

## Methods

A systematic online literature search in PubMed was performed using Boolean logic combining and including the terms “dysphagia,” “swallowing dysfunction,” or “deglutition disorder” and terms reflecting “critical illness” in the titles and excluding malignancies (dysphagia[Title] OR swallowing dysfunction[Title] OR swallowing impair*[Title] OR swallowing disord*[Title] OR deglutition dysfunction[Title] OR deglutition disord* OR deglutition impair*) AND (ICU[Title] OR critical illness[Title] OR intensive care[Title] OR critical care[Title] OR critically ill[Title] OR intubation[Title] OR post-extubation[Title])NOT carcin*[Title] NOT malign*[Title] NOT cancer*[Title] NOT Tumor*[Title] NOT neopla*[Title] NOT palliat*[Title]). In total, 123 articles were included following an initial search strategy using the above stated search string. In detail, *n* = 103 articles were identified by the given search strategy which was followed by a search within the identified articles (identification of an additional *n* = 58 in-article citations). Thirty-eight articles were excluded from the final analysis (*n* = 12 focus on airway and/or anesthetics, *n* = 11 focus not on dysphagia, *n* = 8 pediatric and/or neonatal investigations, *n* = 3 not accessible, *n* = 2 other languages, *n* = 1 neuropsychiatric, *n* = 1 veterinarian investigation). Publications were screened for until December 2018.

### Physiology of swallowing

**Fig. 1:**
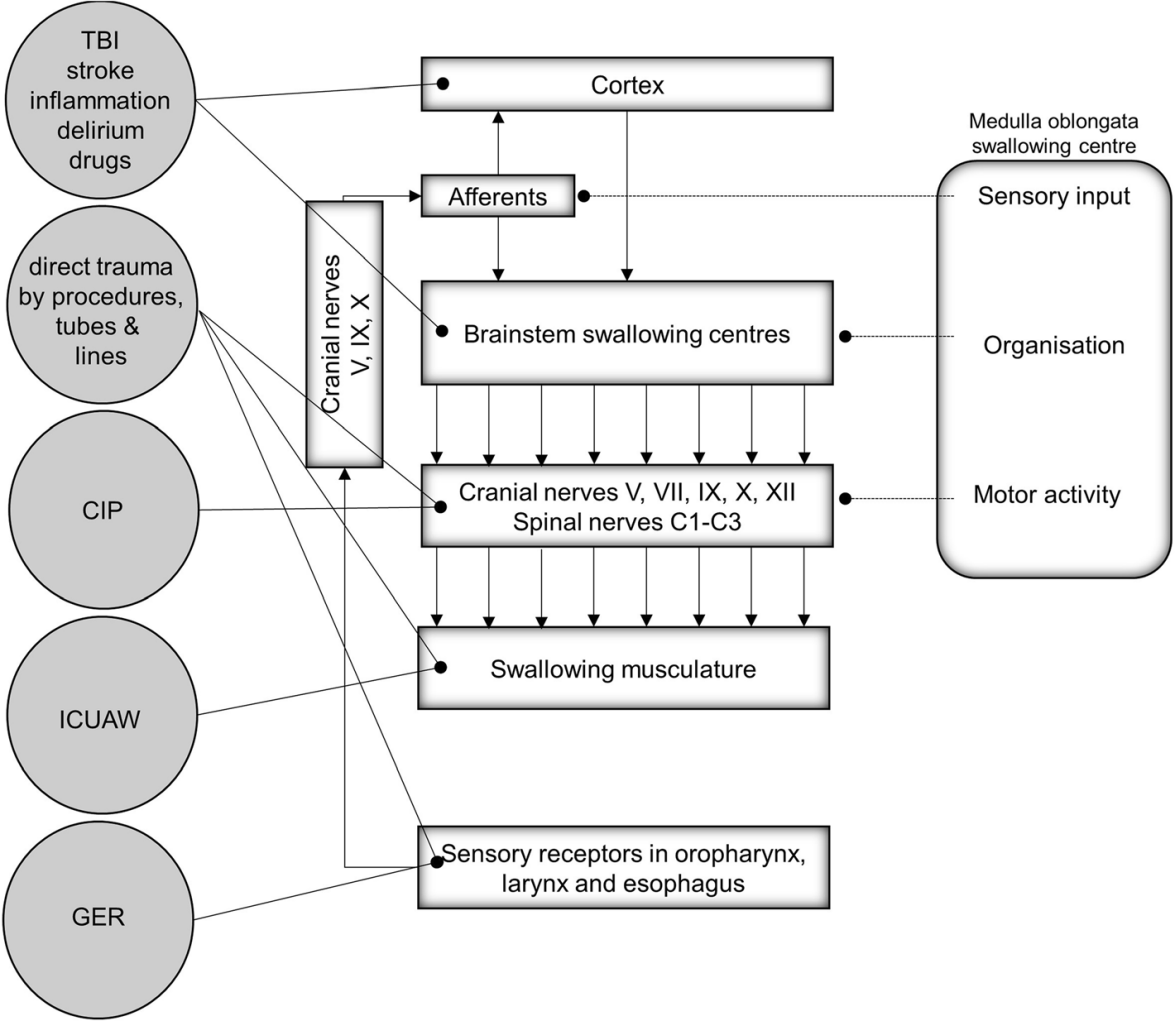
The swallowing network and presumed ICU-related factors for dysphagia. TBI (traumatic brain injury), stroke (ischemic/hemorrhagic), ICUAW (ICU-acquired weakness), GER (gastroesophageal reflux, CIP (critical illness polyneuropathy). Adapted from [139] with permission from the authors, copyright Heike Blum, University Hospital Münster, Germany

**Fig. 2:**
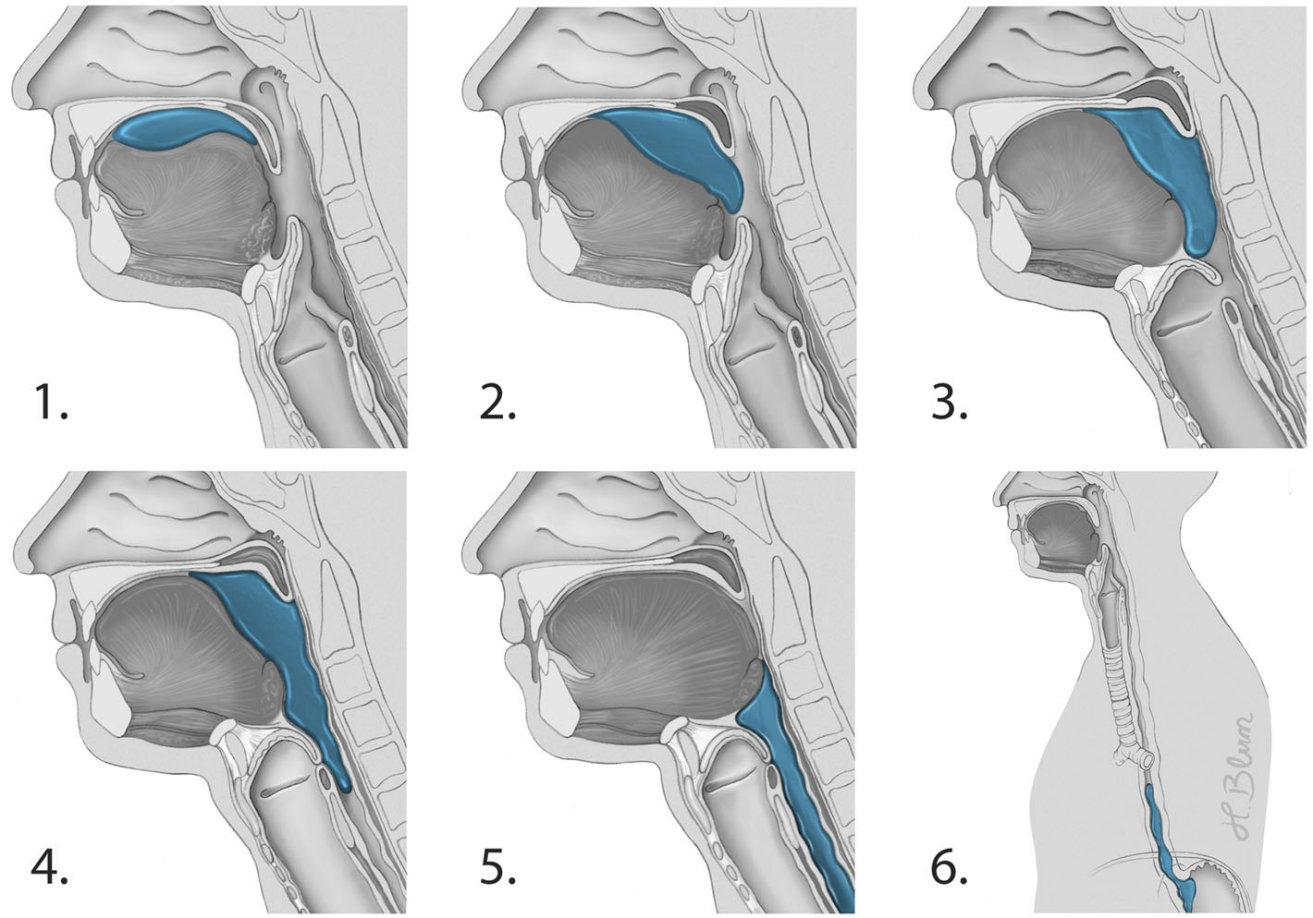
The four stages of swallowing (1 = oral preparatory stage, 2 = oral stage, 3–5 = pharyngeal stage, 6 = esophageal stage). Adapted from [139] with permission from the authors, copyright Heike Blum, University Hospital Münster, Germany

Swallowing is a complex procedure involving more than 50 muscles, and a number of cranial nerves [36] (Fig. 1). Cortical structures involved particularly include the frontoparietal operculum, the primary sensorimotor cortex and association cortices and the anterior part of the insula. Cortico-bulbar projections then target the central pattern generators located in the dorsal medulla oblongata [37–39], and the solitary nucleus and nucleus ambiguous coordinate swallowing [40–42]. In an awake state, individuals swallow involuntarily more than once per minute. The frequency of swallowing is increasing to about > 5 times per minute during meals and decreased to about eight times per hour during sleep [43]. Four phases of swallowing can be differentiated [44]: (1) oral preparatory, (2) oral transit, (3) pharyngeal, and (4) the esophageal phase (Fig. 2). Bolus formation takes place in phase 1. In phase 2, the bolus is placed in the middle of the tongue, pushed against the hard palate and backwards to the oropharynx. When in contact with the palatoglossal arch, the swallowing reflex is triggered and the involuntary pharyngeal phase is initiated. The epipharynx is sealed and airway closure occurs in three different stages: (a) vocal cord adduction, (b) ventricular fold adduction, and (c) contact of arytenoid cartilages with the anteriorly tilted epiglottis. Importantly, active laryngeal elevation occurs which indirectly opens the upper esophageal sphincter. The bolus then enters the epiglottic valleculae and pyriforme sinuses. In the involuntary esophageal phase, peristaltic esophageal waves transport the bolus into the stomach (Fig. 2).

### Pathophysiology of swallowing in critical illness

Oropharyngeal dysphagia in general can be caused by either (1) severe neurological impairment, affecting (a) the central nervous system directly (e.g., stroke, Morbus Parkinson, multiple sclerosis, or amyotrophic lateral sclerosis), (b) due to traumatic peripheral nerve damage and impaired function of the neuro-muscular junction, (c) primary neuro-muscular junction abnormalities (e.g., myasthenia gravis, Lambert-Eaton myasthenic syndrome [LEMS]), or (d) a primary muscular disease (e.g., inflammatory myopathies); (2) structural damage (e.g., trauma caused by the intubation or malignancies); (3) medication or toxic/drug side-effects; (4) presbyphagia; or (5) phagophobia [45].

In critically ill patients on the ICU, the etiology of dysphagia post-extubation appears less clear. PED is considered multifactorial with underlying mechanisms unknown, and the presence of an endotracheal tube/prolonged mechanical ventilation is considered a key risk factor for dysphagia. Six potential key mechanisms for the development of ICU-acquired swallowing disorders including PED were previously suggested [4]: (1) direct trauma caused by endotracheal and tracheostomy tubes, (2) neuromyopathy resulting in muscular weakness, (3) diminished laryngeal sensory function, (4) an impaired sensorium, reflecting a more centrally located problem, (5) gastroesophageal reflux, and (6) dyssynchronous breathing and swallowing.

In detail, direct trauma (1) seems an obvious and major mechanism in ICU-acquired swallowing dysfunction (Fig. 1). Artificial tubes of any kind, e.g., endotracheal, tracheostomy, echocardiography probes, or potentially feeding tubes, could directly cause trauma to anatomic structures. This may especially be relevant in emergency diagnostic or therapeutic interventions. Mechanical irritations of the underlying mucosal tissue may lead to focal ulceration and/or triggering of localized inflammatory processes. Scarring of the vocal cords was reported also, known as “vocal cord synechiae” [46]. Cuffed airway tubes per se prohibit normal swallowing function and active laryngeal elevation and subsequently reduce the passive opening of the upper esophageal sphincter which impedes rapid esophageal passage [45]. Two smaller studies showed contradicting results: One study [47] found no effect on penetration or aspiration by cuff status (inflated vs. deflated), but a significantly reduced score for the liquid bolus when a one-way valve was placed in comparison to in- or deflated cuff conditions (*n* = 14). Another study with a limited sample size (*n* = 7) [48] observed no significant alteration in hyoid bone movement and laryngeal excursion in cases when a tracheotomy tube was placed. Further, long-term intubation may lead to dislocation or even subluxation of arytenoid cartilages, resulting in an impaired glottis closure during swallowing [49]. Further, traumatic laryngoscopy was shown to lead to hypoglossal nerve palsy and to cause dysphagia [50–53]. Peripheral damage of the recurrent laryngeal nerve, e.g., caused by tube cuff compression (or as a complication during surgery) can result in vocal cord paresis/paralysis and may prohibit competent airway protection.

Another relevant aspect is the presence of ICU acquired weakness (ICUAW) [54]. In critically ill patients with ICUAW, general muscular weakness and muscular atrophy was reported, which may affect the swallowing apparatus [54–56]. ICUAW may be a consequence of “disuse” in patients receiving long-term intubation, long-term (analgo)-sedation, and/or neuromuscular blocking agents [28, 54, 57, 58]. Further, specific swallow-related muscular weakness was recently suggested in previously orally intubated acute respiratory distress syndrome patients (*n* = 11, median duration of intubation 14 days) after examination by videofluoroscopic swallowing study (VFSS) [59]. In addition, in ICU-acquired ventilator-induced diaphragmatic dysfunction (VIDD) [55], cough strength could be diminished leading to limited glottic clearance. Importantly, reduced local sensation appears as an additional key problem in ICU-acquired dysphagia. Either caused by direct mechanical damage, local inflammation/edema, or by critical illness polyneuropathy (CIP) [54, 56], afferent sensory pathways may be impaired leading to swallowing dysfunction [60–63]. Clinically, this may become apparent when a bolus reaches the reflex trigger zone in the palatoglossal arch but the afferent input is impaired resulting in a delayed swallow response and pre-deglutitive aspiration. However, the exact role of sensory impairment in critically ill patients appears unclear. In a recent study, no nerve conduction abnormalities were demonstrated, questioning the role of CIP in dysphagia [27].

Central (cerebral) problems in ICU-acquired swallowing disorders are mostly caused by direct damage to the central nervous system, e.g., in traumatic brain injury, stroke/hemorrhage, and/or inflammatory disorders. Contributing to this, reduced qualitative (e.g., in delirium) or quantitative level of consciousness further increases the risk for aspiration [64] and may delay therapeutic measures for dysphagia. Moreover, drug-induced effects (e.g., (analgo-) sedatives or various neurotropic medications) may affect swallowing either centrally (mostly via reduced consciousness) or peripherally (mostly at the neuro-muscular junction). In this context, another potential mechanism was suggested [4], i.e., exact coordination of laryngeal closure, apnea, and opening of the upper esophageal sphincter may be impaired. In critically ill patients, this is referred to as “dyssynchrony” between respiration and swallowing [4]. Furthermore, in critically ill patients with respiratory distress, the apnoeic period during swallowing is shortened with potential premature opening of the larynx before the bolus has passed into the esophagus [65].

### Terminology of dysphagia on the ICU

Different terms are used to assess dysphagia. In the 10th revision of the International Statistical Classification of Disease and Related Health Problems (ICD-10, WHO-Version 2016), dysphagia (R13) is listed in “Chapter XVIII: symptoms, signs and abnormal clinical and laboratory findings,” not elsewhere classified and more specified under R10–19 in “Symptoms and signs involving the digestive system and abdomen.” Dysphagia, swallowing disorder, or deglutition disorder/dysfunction are often used synonymously. In 2013, the term ICU-acquired swallowing disorder was introduced [4] suggesting multiple potential pathomechanisms in critical illness leading to acquired dysphagia in a previously dysphagia-naïve patient. International consensus on dysphagia definitions is lacking which may negatively impact on data comparability. We therefore recently proposed a delphi procedure with the aim to harmonize respective terminology [66].

### Epidemiology of dysphagia on the ICU

A systematic review on the incidence of PED published in 2010 included a total of 14 studies with a total of 3520 individuals (mean of approximately 251 patients per study, median of 67) and concluded that the incidence rate ranges from 3 to 62% [7]. Study design, patient selection (e.g., assessment of patients post-aspiration), and/or limited patient numbers in respective included studies introduced a high risk of bias and showed reduced quality of evidence [1–3, 5, 6, 67]. In a subsequent retrospective observational cohort study, a dysphagia prevalence of up to 84% was reported [67]. A recent larger study (*DYnAMICS*) performed by us included 1304 medical and surgical ICU patients with potential PED risk reported an incidence rate of 12.4% (18.3% in unselected emergency admissions) after systematic screening [8]. In DYnAMICS, the incidence was likely underestimated due to exclusion of patients leaving the ICU alive with tracheostomy (no extubation/decannulation) [8].

### Risk factors for dysphagia on the ICU

Risk factors for dysphagia might theoretically be inferred from the abovementioned pathomechanisms. However, studies focusing on risk factors for dysphagia following endotracheal intubation are scarce and provide conflicting results. Studies are mostly of limited sample size and either supporting or rejecting respective factors, including factors such as age [12, 19, 20, 68–75], decreased cardiac output [19, 70], intubation duration [19, 20, 22, 32, 70, 71, 73, 74], postoperative pulmonary complications [70, 74], tube feeding [19, 22, 32, 74], sepsis [6, 32, 72], transesophageal echocardiogram (TEE) [70, 71], perioperative stroke [3, 32, 69–71], or gastroesophageal reflux [1, 19, 76]. More consistently rejected as potential risk factors are APACHE II and SOFA scores [1, 3, 6, 9, 22, 67]; BMI [1, 6, 9]; gender [1, 6, 9, 32, 70, 75, 77, 78]; comorbidities such as arterial hypertension, kidney disease, diabetes, COPD, myocardial infarction, or heart failure [3, 19, 32, 67, 69, 70, 72], as well as smoking [32, 72]; and endotracheal tube size [3, 67, 75]. These contradicting results reflect bias due to patient selection, differing study/screening protocols, and limited patient numbers. However, despite controversial discussions, it appears that most presumed dysphagia risk factor would be duration of intubation/mechanical ventilation [1, 3, 4, 12, 32, 67, 68, 70, 78–80]. In addition, rather accepted risk factors [4] may include the presence of pre-existing dysphagia, local malignancy/post-surgical medical conditions affecting anatomic structures of the swallowing tract, and/or considerable quantitative/qualitative reduction of consciousness. Overall, large-scale clinical data is missing and strongly warranted in order to potentially reduce the number of patients affected by preventional measures.

### Assessment of dysphagia in the critically ill

In stroke patients, early dysphagia detection minimizes the risk of aspiration [81] and systematic dysphagia screening reduces stroke-associated pneumonia rates [82]. This suggests that a systematic routine screening approach should be performed in all patients at risk without limitation to selected patient cohorts (e.g., stroke patients). Non-instrumental [66] and instrumental measures are available for timely assessment of dysphagia in the critically ill [83]. Non-instrumental assessments for dysphagia are typically performed by trained specialists (e.g., speech-language therapists, physiotherapists, or occupational therapists). The following clinical examinations were previously proposed for general populations of hospitalized (mostly non-ICU) patients: the bedside swallowing evaluation (BSE) [84], the Volume Viscosity Swallowing Test (V-VST) [85], the Mann Assessment of Swallowing Ability (MASA, K-MASA, MASA-C, MMASA) [86–89], the McGill Ingestive Swallowing Assessment (MISA, MISA-DK) [90, 91], the Gugging Swallowing Screen (GUSS) [92], the Northwestern Dysphagia Patient Check Sheet (NDPCS) [93], the Dysphagia Disorder Survey (DDS) [94], the Practical Aspiration Screening Scheme (PASS) [95], the Kuchi-Kara Taberu Index (KT Index) [96], and the Practical Assessment of Dysphagia [97] test (reviewed in [66]).

Instrumental tests, such as the flexible endoscopic evaluation of swallowing (FEES) or the VFSS, may be regarded the gold standard of dysphagia assessment in the critically ill. FEES can be performed at the ICU bed using a small flexible endoscope passing through a nostril into the epipharynx so that the oro-/ hypopharynx and the glottic area can be visualized. Using a multicolor dye technique [98], testing of different food consistencies can be performed. Further, in selected patients (depending upon availability), sensation testing can be performed using short blasts of air to the supraglottic mucous membrane for assessment of vocal cord adduction, a technique known as flexible endoscopic evaluation of swallowing with sensory testing (FEESST) [99, 100]. Apart from research, FEESST was mainly abandoned and replaced by touching the aryepiglottic area with the endoscope tip, rendering the sensation normal, absent, or reduced. In FEES, severity of penetration or aspiration is assessed using a penetration and aspiration (PAS) scale (1 indicating no penetration and 8 indicating aspiration without coughing, i.e., silent aspiration) [101]. VFSS, also referred to as “modified barium swallow,” requires patient transfer to a radiology suite, which limits feasibility in larger cohorts of critically ill patients. Although exposure to radiation may be a disadvantage, VFSS investigates the entire swallowing act, i.e., all four stages of swallowing [102, 103]. Different barium-containing food consistencies can be visualized and recorded using high-resolution imaging devices. Intra-deglutitive aspiration can be visualized. This is not possible in FEES due to a “white out”-effect caused by velum elevation. Besides providing proof for the diagnosis, effects of compensatory maneuvers and diet modifications can be studies in a real-time manner using VFSS or FEES. Further “instrumental” methods include ultrasonography [104], tissue Doppler imaging [105], high-resolution manometry [106–110], and oropharyngo-esophageal scintigraphy (OPES) [111]. Whereas manometry can be used to assess pharyngeal propulsion and upper esophageal sphincter performance, OPES allows detailed analysis of transit times and potential retention of a food bolus in the various anatomical areas. However, in critically ill patients post-extubation, this appears not feasible.

We recently proposed a feasible, pragmatic approach for systematic dysphagia assessment in the ICU [8]. This includes a two-step approach with systematic bedside screening for dysphagia by trained ICU nurses within few hours post-extubation, followed by an expert exam that is optimally complemented by a confirmatory FEES investigation [8].

### Clinical consequences of dysphagia in the critically ill

In the critically ill, only few studies analyzed the impact of PED on patient-centered clinical outcomes. A recent retrospective study found an independent association of dysphagia with a composite endpoint of pneumonia, reintubation, or death [67]. In addition, dysphagia was associated with longer hospitalization, more discharges to a nursing home, and increased need for placement of a feeding tube [67, 77]. In a recent large prospective observational study (DYnAMICS) in a mixed population of 1304 critically ill patients with systematic bedside screening for dysphagia post-extubation, we observed an independent association of PED with 28-day and 90-day mortality after adjustment for typical confounders. An excess of 9.2% of a 90-day mortality rate was observed in patients with dysphagia [8].

In summary, the clinical consequences of dysphagia in the critically ill are important, with prolonged length of hospitalization, increased resource use, increased treatment costs, and increased mortality [8, 31, 67, 112]. Early identification of patients at risk seems warranted in an effort to minimize respective burdens.

### Therapeutic considerations

#### Overview

The body of evidence for dysphagia treatment, especially in dysphagia-positive ICU patients, is limited. Generally, three major therapeutic pillars for dysphagia treatment are considered [113]: dietary texture modifications, postural changes/compensatory maneuvers, and interventions aiming to improve swallowing function (e.g., devices using neuromuscular stimulation).

#### Dietary texture modification and compensatory maneuvers

Adaptation, a term mostly used in the German-speaking literature [45], is referring to as texture modification according to the deglutition pathology and use of technical aids, e.g., prosthetics to account for velopharyngeal defects, in an effort to optimize swallowing. Compensation is referring to either compensatory maneuvers and/or postural changes to address swallowing deficiencies. Special swallowing techniques, e.g., supraglottic swallowing, may support patients with delayed swallowing reflex or incomplete laryngeal closure (e.g., after cordectomy). The patient would then be trained to hold the breath before and during swallowing and forced to cough immediately afterwards to optimize glottic/throat clearance [114, 115]. For patients with impaired laryngeal elevation, reduced tongue force, or dysfunctional opening of the upper esophageal sphincter (UES), the Mendelsohn maneuver can be applied. In this maneuver, during oral preparatory stage, the patient presses the bolus as forcefully as possible against the hard palate for up to 3 s. This elevates the larynx and improves UES opening and clearance of food residuals [116]. Postural change “chin down” reduces the distance between to the tongue basis and pharyngeal dorsal wall, hence narrowing the airway and therefore reducing the risk for leaking (bolus enters prematurely the pharynx) or aspiration within patients known for a delayed swallowing reflex. Moreover, the epiglottic vallecula gets distended, facilitating esophageal bolus passage [117–119].

Head movements (backwards, lateralization towards/away to the side of the paresis/palsy) may also be useful in transporting the bolus to the swallow reflex trigger area [117] or facilitating bolus passage via the healthy piriform recess [117, 120]. Functional dysphagia therapy was found successful in improving swallowing function in patients suffering from neurogenic dysphagia [121]. Apparently, an intensive therapy approach with five trainings a week seems to be more effective in the acute stage of swallowing dysfunction [122]. In addition, sphincter myotomy is an irreversible option in patients with a functionally obstructing of the upper esophageal sphincter to facilitate pharyngo-esophageal bolus propulsion [123, 124]. Medialization thyreoplasty can further be applied in patients with unilateral vocal cord paresis and suffering from aspiration to improve cough and throat clearance [125]. A laryngectomy poses a last resort for patients with persisting aspiration and suffering from repeated severe consequences. In doing this, breathing and alimentary pathways become completely separated.

#### Interventional/technological approaches: pharyngeal electrical stimulation

**Fig. 3:**
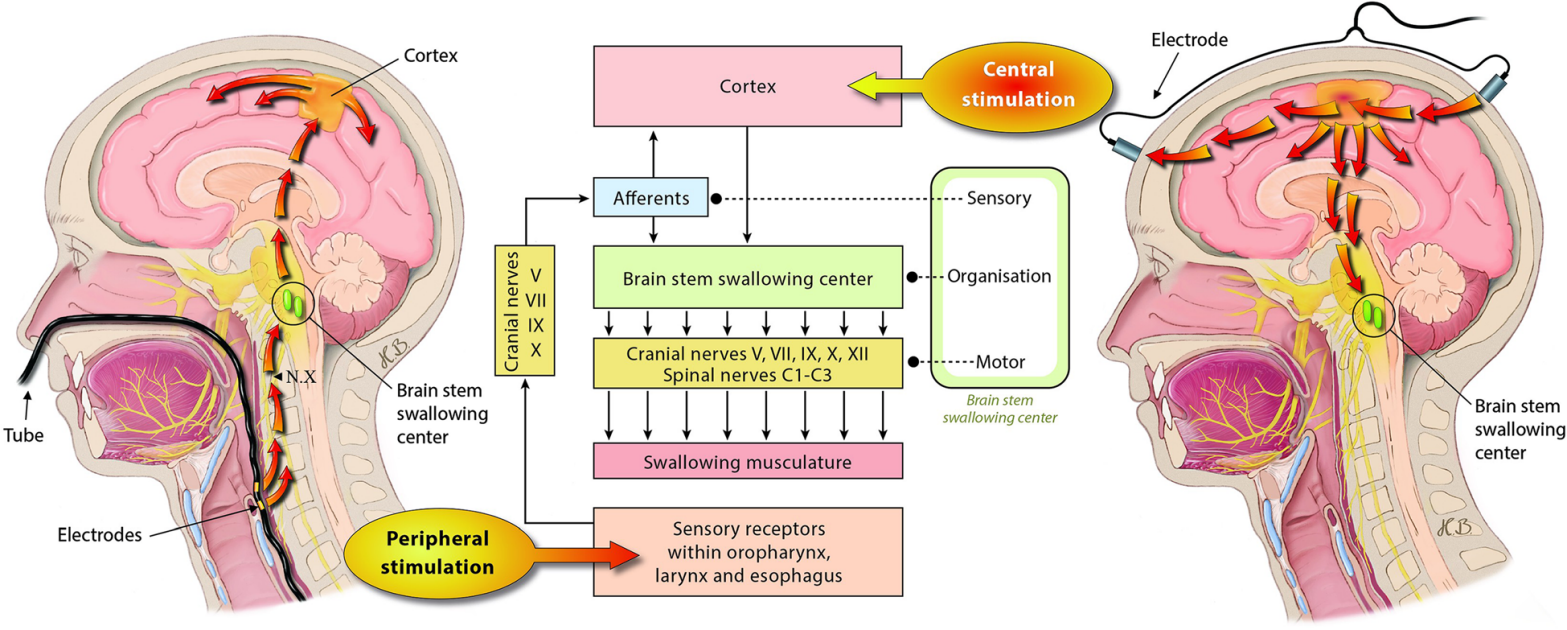
Peripheral (pharyngeal electrical stimulation, PES) and central (transcranial direct current stimulation, tDCS) stimulation strategies targeting the swallowing network. Reprinted with permission from the authors [140], copyright Heike Blum, University Hospital Münster, Germany

Recently, pharyngeal electrical stimulation (PES) (Fig. 3) was proposed as a novel treatment modality using a gastric feeding tube-like stimulation catheter to enhance neuromuscular pharyngeal stimulation, targeted to the individual patient. Stimulation levels are personalized at the start of the treatment to ensure that optimal levels of stimulation are delivered. PES is considered to target the afferent sensory feedback within the swallowing network that seems crucial for swallowing safety and efficacy of motor execution [126, 127]. PES may involve two postulated key modes of action: (a) facilitation of cortico-bulbar pathways [128] and (b) increase of swallowing processing efficiency in respective central nervous system areas [129], e.g., the right primary and secondary sensorimotor cortex and the right supplementary motor area. Data also demonstrate an increase of pharyngeal cortical representation and motor excitability for more than half an hour after 10 min of PES treatment. A dose-response study ([130]) showed optimal cost-effectiveness when applying a PES protocol with one cycle of 10-min stimulation per day for a total of 3 consecutive days. Further, substance P is known to enhance the swallow and cough reflex [131, 132] with reduced amounts of being observed in the sputum of elderly people suffering from aspiration pneumonia [133]. As demonstrated [134], PES may induce local substance P release into the saliva. This peripheral action with a postulated local sensitization of primary sensory neurons in the oropharyngeal effector area may then facilitate a motor swallow response by the cerebral cortex in a remote fashion.

Recently, PES was shown of therapeutic potential, especially within selected patient populations, i.e., in patients with post-stroke dysphagia (PSD). In a cohort of patients with severe persisting PSD with tracheostomy and decannulation failure, a recent study could demonstrate enhanced remission of dysphagia resulting in decannulation in 75% of PES-treated patients vs. 20% of patients decannulated in sham-treated individuals [129]. Two recently published studies support respective findings [135, 136]. Due to its ease of application, PES seems to be a suitable treatment approach for daily clinical practice in patients with post-stroke dysphagia. PES was validated in stroke patients, and data support a reduced hospital length of stay after treatment with PES [136].

### Outlook

In the light of the fact that dysphagia is not routinely screened for in critically ill patients on most ICUs, it appears that post-extubation dysphagia awareness should be increased [137, 138] and systematic bedside screening should be implemented on the ICU [8]. Further, after identification of patients with PED on the ICU, rehabilitation measures should be started. The promising results of recent randomized controlled trials assessing PES, rTMS (repetitive transcranial magnetic stimulation), and/or tDCS (transcranial direct current stimulation) (Fig. 3) pointed to positive effects in patients with severe post-stroke dysphagia. In the future, a novel therapeutic interventions using peripheral afferent approaches (e.g., via PES) and/or central efferent stimulation of pathways of the swallowing network may be of particular interest.

## Conclusions

In the light of the fact that the clinical consequences of ICU-acquired dysphagia (e.g., aspiration-induced pneumonitis/pneumonia) can often be observed on ICUs, more data on underlying mechanisms and/or risk factors seems required. Post-extubation dysphagia as a key subgroup affects a considerable number of critically ill patients and often persists far beyond ICU discharge. Awareness for dysphagia on the ICU should be increased, and systematic screening protocols should be established. Furthermore, dysphagia on the ICU appears an overlooked health care problem and studies on novel therapeutic interventions seem warranted.
